# Metabolic Engineering of *Bacillus subtilis* Toward Taxadiene Biosynthesis as the First Committed Step for Taxol Production

**DOI:** 10.3389/fmicb.2019.00218

**Published:** 2019-02-20

**Authors:** Ingy I. Abdallah, Hegar Pramastya, Ronald van Merkerk, Sukrasno, Wim J. Quax

**Affiliations:** ^1^ Department of Chemical and Pharmaceutical Biology, Groningen Research Institute of Pharmacy, University of Groningen, Groningen, Netherlands; ^2^ Pharmaceutical Biology Research Group, School of Pharmacy, Institut Teknologi Bandung, Bandung, Indonesia

**Keywords:** *Bacillus subtilis*, metabolite, MEP, GGPPS, taxadiene, Taxol

## Abstract

Terpenoids are natural products known for their medicinal and commercial applications. Metabolic engineering of microbial hosts for the production of valuable compounds, such as artemisinin and Taxol, has gained vast interest in the last few decades. The Generally Regarded As Safe (GRAS) *Bacillus subtilis* 168 with its broad metabolic potential is considered one of these interesting microbial hosts. In the effort toward engineering *B. subtilis* as a cell factory for the production of the chemotherapeutic Taxol, we expressed the plant-derived taxadiene synthase (TXS) enzyme. TXS is responsible for the conversion of the precursor geranylgeranyl pyrophosphate (GGPP) to taxa-4,11-diene, which is the first committed intermediate in Taxol biosynthesis. Furthermore, overexpression of eight enzymes in the biosynthesis pathway was performed to increase the flux of the GGPP precursor. This was achieved by creating a synthetic operon harboring the *B. subtilis* genes encoding the 2-C-methyl-D-erythritol-4-phosphate (MEP) pathway (*dxs*, *ispD*, *ispF*, *ispH*, *ispC*, *ispE*, *ispG*) together with *ispA* (encoding geranyl and farnesyl pyrophosphate synthases) responsible for providing farnesyl pyrophosphate (FPP). In addition, a vector harboring the *crtE* gene (encoding geranylgeranyl pyrophosphate synthase, GGPPS, of *Pantoea ananatis*) to increase the supply of GGPP was introduced. The overexpression of the MEP pathway enzymes along with IspA and GGPPS caused an 83-fold increase in the amount of taxadiene produced compared to the strain only expressing TXS and relying on the innate pathway of *B. subtilis*. The total amount of taxadiene produced by that strain was 17.8 mg/l. This is the first account of the successful expression of taxadiene synthase in *B. subtilis*. We determined that the expression of GGPPS through the *crtE* gene is essential for the formation of sufficient precursor, GGPP, in *B. subtilis* as its innate metabolism is not efficient in producing it. Finally, the extracellular localization of taxadiene production by overexpressing the complete MEP pathway along with IspA and GGPPS presents the prospect for further engineering aiming for semisynthesis of Taxol.

## Introduction

Terpenoids represent the largest, structurally and functionally most varied group of natural products. This diversity is based on a structural complexity that cannot be simply reproduced using chemical synthetic processes. Nowadays, there are over 50,000 known terpenoids, a lot of which are biosynthesized by plants. Numerous terpenoids have attracted commercial interest for their medicinal value or use as flavors and fragrances. Among the most famous medicinally important terpenoids are the antimalarial artemisinin from the plant *Artemisia annua* and the anticancer paclitaxel (Taxol^®^) from the yew trees (*Taxus brevifolia* or *Taxus baccata*). The majority of terpenoids are naturally produced in low amounts and their extraction is usually labor intensive, and it entails considerable consumption of natural resources. For instance, the production of enough Taxol^®^ to treat one cancer patient would approximately require six 100-year-old Pacific yew trees, and similarly, there are reports of enormous shortfalls in artemisinin production due to seed shortage. In addition, chemical synthesis and modification of most terpenoids is tremendously difficult and problematic because of the complexity and chirality of their chemical structures. Hence, researchers in the last few decades focused on metabolic engineering of the terpenoid biosynthetic pathways in host microorganisms as an alternate method of production (Connolly and Hill, 1991; Wagner et al., 2000; Koehn and Carter, 2005; Julsing et al., 2006; Klein-Marcuschamer et al., 2007).

The backbone of all terpenoids originates from 2 five-carbon precursors, isopentenyl diphosphate (IPP) and its isomer dimethylallyl diphosphate (DMAPP), which can be produced *via* the mevalonate (MVA) pathway or the 2-C-methyl-D-erythritol-4-phosphate (MEP) pathway. The consecutive condensation of IPP and DMAPP catalyzed by a group of prenyl pyrophosphate synthase enzymes produces the starting precursors of the different classes of terpenoids. These are (1) geranyl pyrophosphate (GPP; C_10_) produced by geranyl pyrophosphate synthase (GPPS) for the synthesis of monoterpenoids, (2) farnesyl pyrophosphate (FPP; C_15_) produced by farnesyl pyrophosphate synthase (FPPS) for the construction of sesquiterpenoids and triterpenoids, and (3) geranylgeranyl pyrophosphate (GGPP; C_20_) synthesized by geranylgeranyl pyrophosphate synthase (GGPPS) for the production of diterpenoids and tetraterpenoids. Finally, these starting precursors are cyclized and/or rearranged by terpene synthase enzymes to yield the different terpenoids (Withers and Keasling, 2007; Muntendam et al., 2009; Abdallah and Quax, 2017).

**Figure 1:**
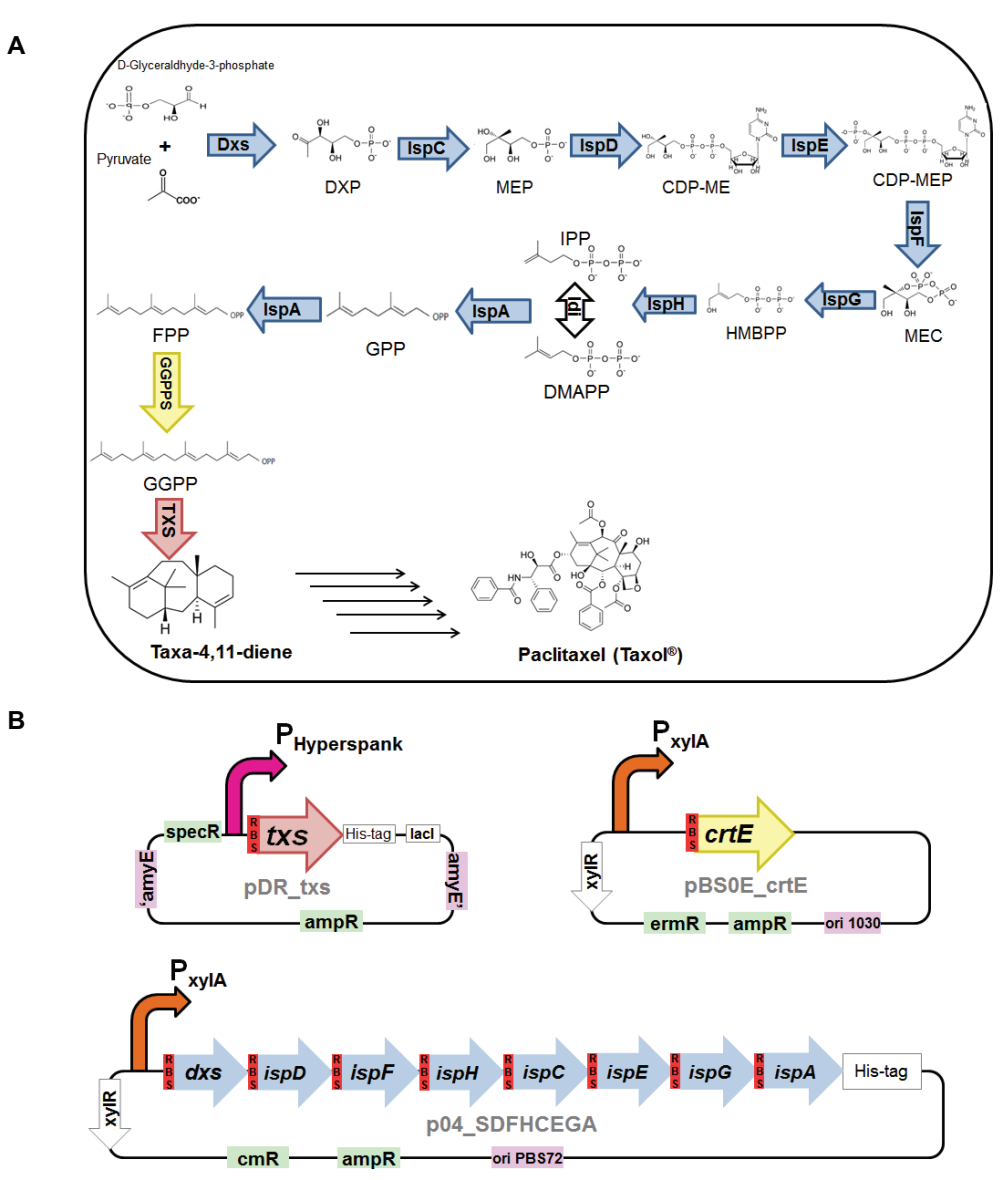
**(A)** Biosynthesis of taxa-4,11-diene *via* the 2-C-methyl-D-erythritol-4-phosphate (MEP) pathway in *Bacillus subtilis*. **Intermediates in the metabolic pathway:** 1-deoxy-D-xylulose 5-phosphate (DXP), 2-C-methyl-D-erythritol 4-phosphate (MEP), 4-(cytidine 5′-diphospho)-2-C-methyl-D-erythritol (CDP-ME), 2-phospho-4-(cytidine 5′-diphospho)-2-C-methyl-D-erythritol (CDP-MEP), 2-C-methyl-D-erythritol 2,4-cyclodiphosphate (MEC), (E)-4-hydroxy-3-methylbut-2-en-1-yl diphosphate (HMBPP), isopentenyl diphosphate (IPP), dimethylallyl diphosphate (DMAPP), geranyl pyrophosphate (GPP), farnesyl pyrophosphate (FPP), and geranylgeranyl pyrophosphate (GGPP). **Enzymes in the biosynthesis pathway:** 1-deoxy-D-xylulose-5-phosphate synthase (Dxs), 1-deoxy-D-xylulose-5-phosphate reductoisomerase, or 2-C-methyl-D-erythritol 4-phosphate synthase (Dxr, IspC), 2-C-methyl-D-erythritol 4-phosphate cytidylyltransferase (IspD), 4-(cytidine 5′-diphospho)-2-C-methyl-D-erythritol kinase (IspE), 2-C-methyl-D-erythritol 2,4-cyclodiphosphate synthase (IspF), (E)-4-hydroxy-3-methylbut-2-enyl-diphosphate synthase (IspG), 4-hydroxy-3-methylbut-2-enyl diphosphate reductase (IspH), isopentenyldiphosphate delta-isomerase (Idi), IspA which act as geranyl pyrophosphate synthase (GPPS) and farnesyl pyrophosphate synthase (FPPS), geranylgeranyl pyrophosphate synthase (GGPPS), and taxadiene synthase (TXS). **(B)** Plasmid constructs used for engineering *B. subtilis*. pDR_txs contains *txs* gene (red), preceded with *B. subtilis mntA* ribosomal binding site (dark red), to be inserted into the genome of *B. subtilis* between the *amyE* front flanking region and *amyE* back flanking region (purple), IPTG inducible hyperspank promoter (pink), and ampicillin and spectinomycin resistance cassettes (green). pBS0E_crtE contains *crtE* gene (yellow) encoding for GGPPS and preceded with *B. subtilis mntA* ribosomal binding site (dark red), xylose inducible promoter (orange), and ampicillin and erythromycin resistance cassettes (green). p04_SDFHCEGA contains seven genes of the MEP pathway, *dxs*, *ispD*, *ispF*, *ispH*, *ispC*, *ispE*, and *ispG*, along with the gene *ispA* (blue), each preceded with *B. subtilis mntA* ribosomal binding site (dark red), in a synthetic operon controlled by xylose inducible promoter (orange) and ampicillin and chloramphenicol resistance cassettes (green).

Paclitaxel (Taxol^®^) is a diterpenoid known for its chemotherapeutic effect and is found in the bark and needles of different *Taxus* trees. Similar to all terpenoids, the extraction from the natural source is problematic, thus various *Taxus* species are now endangered due to high demand. Total synthesis of paclitaxel has been established, but the complexity of its chemical structure made the process commercially inapplicable (Nicolaou et al., 1994). Hence, nowadays paclitaxel is synthesized semisynthetically from 10-deacetylbaccatin III that is more easily extracted from *Taxus* needles. Also, docetaxel, which has been gaining more attention recently due to its higher water solubility leading to improved pharmacokinetic properties and better potency, can be synthesized from this precursor. However, this means that production still relies on the yew trees (Wuts, 1998; Baloglu and Kingston, 1999; Dewick, 2001). The first step in the production of paclitaxel is the production of the compound taxa-4,11-diene (Figure 1A). Taxadiene is produced from the cyclization of the diterpenoid precursor GGPP *via* the enzyme taxadiene synthase. The GGPP precursor can be synthesized *via* the MVA and/or the MEP pathway as previously explained. Taxadiene is converted to the final product, paclitaxel, through approximately 19 enzymatic steps involving hydroxylation and other oxygenation reactions of the taxadiene skeleton (Hezari and Croteau, 1997; Julsing et al., 2006; Abdallah and Quax, 2017).

The first committed intermediate in biosynthesis of paclitaxel, taxadiene, has been produced *via* metabolic engineering in *Escherichia coli* (Huang et al., 2001; Ajikumar et al., 2010), *Saccharomyces cerevisiae* (DeJong et al., 2006; Engels et al., 2008), and the transgenic plant *Arabidopsis thaliana* (Besumbes et al., 2004). Based on the success of taxadiene production in these hosts, *Bacillus subtilis* represents an interesting microbial host for the production of taxadiene where it has higher growth rate compared to *S. cerevisiae* and is mostly considered as GRAS (Generally Regarded As Safe) by the Food and Drug Administration unlike *E. coli* (Zhou et al., 2013). Also, *B. subtilis* possesses an innate MEP pathway that can be manipulated to increase the flux of precursors. An optimally regulated synthetic operon encompassing MEP pathway genes has been reported to lead to a high production of C_30_ carotenoids in *B. subtilis* (Xue et al., 2015). Also, the sesquiterpenoid amorphadiene, which is the first precursor for the production of artemisinin, has been successfully produced in *B. subtilis* (Zhou et al., 2013). In the current study, we aim at the metabolic engineering of *B. subtilis* for the biosynthesis of taxadiene as a first step in the semisynthetic production of paclitaxel. For the first time, we describe the successful expression of the enzyme taxadiene synthase essential for the synthesis of taxadiene in *B. subtilis*. Moreover, the production levels of taxadiene were increased by overexpression of the MEP pathway, IspA, and GGPPS enzymes. The reported *B. subtilis* strain with the highest level of production of taxadiene can strive to exceed yeast and *E. coli*, besides having the additional advantages provided from the use of *B. subtilis*. This study can serve as a stepping stone for further fine tuning of the biosynthetic pathway of paclitaxel in *B. subtilis* targeted at a sustainable and efficient production process.

## Materials and Methods

### Bacterial Strains, Vectors, and Growth Conditions

Bacterial strains and expression vectors used in this research are listed in Table 1. *E. coli* DH5α strains were cultured in Luria-Bertani broth (LB), while *B. subtilis* 168 strains were grown in 2xYT medium. When necessary, growth media were supplemented with antibiotics in the following concentrations: 100 μg/ml ampicillin or 100 μg/ml erythromycin for *E. coli* DH5α and 5 μg/ml chloramphenicol, 10 μg/ml erythromycin, or 10 μg/ml spectinomycin for *B. subtilis* 168.

**Table 1:** Bacterial strains and vectors used in this research.

**Bacterial strain**	**Genotype**	**Reference**
*B. subtilis* 168	*trp*C2	(Kunst et al., 1997; Barbe et al., 2009)	
*B. subtilis* 168_txs	168 *amyE*::P*_hyperspank_*-*txs*; Sp^R^	This study	
*E. coli* DH5α	F^−^ *end*A1 *hsd*R17 (r_k_^−^,m_k_^+^) *sup*E44 *thi* ^−^1 λ^−^ *rec*A1 *gyr*A96 *rel*A1 φ80d*lac*Z∆M15	Bethesda Research Lab 1986	
**Vector**	**Pertinent properties**	**Reference**
pDR111	*B. subtilis* integration vector; ori-pBR322; P*_hyperspank_* IPTG-inducible promoter; Sp^R^; Amp^R^	(Overkamp et al., 2013)
pBS0E	*B. subtilis* and *E. coli* shuttle vector; ori-1030 (theta replication); copy number ~15–25; P*_xylA_* xylose-inducible promoter; Erm^R^; Amp^R^	(Popp et al., 2017)
pHCMC04G	*B. subtilis* and *E. coli* shuttle vector; ori-pBS72 (theta replication); copy number ~6; P*_xylA_* xylose-inducible promoter; Cm^R^; Amp^R^	(Xue et al., 2015)

### Construction of Different Strains of *B. subtilis* 168

Three different constructs were utilized to produce different *B. subtilis* strains expressing taxadiene synthase (TXS). The first construct consists of *txs* gene from the plant *Taxus baccata* in pDR111 plasmid. The *txs* gene was truncated by deleting the first 60 amino acids to remove the signal peptide targeting the plastid in order to improve expression and solubility of the protein (Williams et al., 2000). Circular polymerase extension cloning (CPEC) (Quan and Tian, 2011) was used to create the pDR_txs construct (Figure 1B) where *B. subtilis mntA* ribosomal binding site (RBS) plus spacer (AAGAGGAGGAGAAAT) were introduced before the *txs* gene along with a N-terminal 6× His-tag. Ampicillin and spectinomycin resistance cassettes are available for selection in *E. coli* and *B. subtilis*, respectively. The expression of TXS is controlled by IPTG inducible hyperspank promoter. The second construct consists of the *crtE* gene, encoding the GGPPS of *Pantoea ananatis*, together with the *mntA* RBS preceding the coding region. The gene was cloned into pBS0E plasmid containing xylose inducible promoter using CPEC method resulting in the construct pBS0E_crtE (Figure 1B). Finally, a construct expressing MEP pathway genes in pHCMC04G plasmid was used. The construct p04_SDFHCEGA (Figure 1B) expresses all the seven genes of the MEP pathway, *dxs*, *ispD*, *ispF*, *ispH*, *ispC*, *ispE*, and *ispG*, along with the gene *ispA*, each with its own engineered RBS, in one synthetic operon controlled by a xylose inducible promoter. p04_SDFHCEGA (16.4 Kb) was cloned using previously available constructs, p04_SDFH and p04_CEGA, by applying circular polymerase extension cloning (CPEC) (Quan and Tian, 2011; Xue et al., 2015). All these cloning steps were performed in *E. coli* DH5α, and the sequences of all the generated recombinant plasmids were confirmed by sequencing (Macrogen, Europe). The constructed plasmids were used to transform competent *B. subtilis* 168 cells following previously published protocol (Kunst and Rapoport, 1995). Three different strains of *B. subtilis* 168 were produced by transforming different combinations of the constructs.

### Expression of Taxadiene Synthase in *B. subtilis* 168

Overnight culture of the *B. subtilis* strain containing pDR_txs construct was grown in 2xYT medium containing spectinomycin antibiotic. The next day, the overnight culture was diluted to an OD_600_ of 0.07–0.1 in 10 ml 2xYT medium with spectinomycin in 100-ml Erlenmeyer flask. The culture was incubated for 3 h at 37°C and 220 rpm till OD_600_ of 0.7–0.9. Then, IPTG was added to a final concentration of 1 mM to start induction. The culture was divided into 1 ml cultures in 15 ml round bottom tubes and then grown overnight at 37, 30, or 20°C and 220 rpm. The following day, certain volumes of the cultures normalized to the OD_600_ = 1 were pelleted by centrifugation for 10 min at 11,000 rpm, 4°C and then resuspended in Birnboim lysis buffer (25 mM Tris-HCl, pH 8.0, 50 mM glucose, cOmplete^™^ Protease Inhibitor Cocktail tablet from Sigma, and 25 mg/ml lysozyme) using an amount of 5 ml buffer per 1 g pellet and incubated at 37°C for 30 min. The soluble protein fractions were obtained by centrifugation for 15 min at 11,000 rpm. The total protein concentration in each soluble protein fraction was estimated using NanoDrop^®^ spectrophotometer. The samples from the expression experiment at different temperatures were loaded with the same total protein concentration on NuPAGE® gels (Invitrogen) for separation by SDS-PAGE and then analyzed by Western blotting where mouse peroxidase-conjugated anti-His antibody (catalog no. A7058; Sigma) was used followed by visualization using Amersham ECL Prime Western blotting detection reagent (catalog no. RPN2232; GE Healthcare).

### Production and Extraction of Taxadiene in *B. subtilis* 168

All strains of *B. subtilis* 168 were grown with the suitable antibiotics using the abovementioned protocol for expression. In addition to IPTG, xylose was added at OD_600_ of 0.7–0.9 to a final concentration of 1% to start induction of pBS0E and pHCMC04G constructs in txs + crtE and txs + crtE + SDFHCEGA *B. subtilis* strains. After which the cultures were divided into 1 ml cultures in 15 ml round bottom tubes and overlaid with 100 μl dodecane containing 10 μl of 700 μM *β*-caryophyllene as internal standard and then grown overnight (24 h) at 37, 30, or 20°C with 220 rpm shaking. The dodecane layer was added to trap the taxadiene released by the cells. The 24-h time point was chosen to ensure maximum production of taxadiene (Zhou et al., 2013). The following day OD_600_ of all 1 ml cultures was measured. A comparison between taxadiene production at the different incubation temperatures along with the difference between direct extraction of taxadiene from the culture and extraction of taxadiene from cell lysate was performed. First, extraction without lysis was performed by adding 200 μl hexane to the 1 ml cultures overlaid with dodecane (1:3 dilution of dodecane), and then the cultures were centrifuged for 10 min at 11,000 rpm to separate the aqueous and organic phases. The dodecane-hexane layer was extracted for GC-MS analysis. Secondly, lysis followed by extraction was performed by addition of 100 μl lysis buffer (50 mM Tris-HCl, pH 8.0, 70 mM NaCl, 10 mM MgCl_2_, 25 mg/ml lysozyme, and 0.1 mg/ml Dnase) to the 1 ml cultures overlaid with dodecane and incubation at 37°C for 30 min. Then, 200 μl 12% SDS (sodium dodecyl sulfate) was added. Finally, 200 μl hexane was mixed (1:3 dilution of dodecane), and the cultures were centrifuged for 10 min at 11,000 rpm. The dodecane-hexane layer was extracted for GC-MS analysis and comparison to the extract without lysis. All cultures and extractions were performed in triplicates.

### Quantification of the Sampled Taxadiene

The dodecane-hexane samples were analyzed on an HP-5MS (5% Phenyl)-methylpolysiloxane column (Agilent J&W 0.25 mm inner diameter, 0.25 μm thickness, 30 m length) in a Shimadzu GCMS-QP5000 system equipped with a 17A gas chromatograph (GC) and AOC-20i autoinjector. The samples (2 μl) were injected splitless onto the GC column, and helium was used as the carrier gas. The injector temperature was 250°C, and the oven initial temperature was 100°C with an increase of 15°C per minute up to 130°C and then 5°C per minute till 210°C. After 210°C was reached, the temperature was raised to 280°C with an increase of 35°C per minute and held for 2 min. The solvent cutoff was 8 min. The MS instrument was set to selected ion mode (SIM) for acquisition, monitoring *m*/*z* ion 122 for taxadiene and *β*-caryophyllene. The chromatographic peak areas for taxadiene and *β*-caryophyllene were determined using the integration tools in GCMSsolution 1.20 software (Shimadzu, Den Bosch, The Netherlands). A calibration curve of standard *β*-caryophyllene with concentration range of 0.5–28 mg/L was created based on chromatographic peak areas in SIM mode (*m*/*z* ion 122). For quantification of taxadiene, the peak area for each sample was corrected by using the peak corresponding to the internal standard *β*-caryophyllene (i.e., by multiplication of the taxadiene peak area for the sample by the peak area of reference *β*-caryophyllene, then division by the *β*-caryophyllene peak area of the sample). This correction is to avoid errors due to injection or loss during extraction. The taxadiene concentration in the diluted dodecane sample was calculated by applying the linear regression equation resulting from the calibration curve to each adjusted taxadiene peak area. Finally, the dilution factor was applied to calculate the concentration of taxadiene in the neat dodecane phase, and then this value was divided by 10 to determine the amount of taxadiene produced per liter of culture as the dodecane layer constitutes a second phase (10%) in the culture. The taxadiene concentration obtained for each sample was divided by the OD_600_ of the corresponding culture to calculate the specific taxadiene production value (mg/L/OD_600_) *β*-caryophyllene equivalent (Rodriguez et al., 2014).

### Analysis of Segregational Stability of the Constructs in txs + crtE + SDFHCEGA *B. subtilis* Strain

Segregational stability was measured by evaluating the growth of *B. subtilis* 168 strain harboring the pDR_txs, pBS0E_crtE, and p04_SDFHCEGA constructs in 2xYT medium without antibiotics for 30 generations involving several subcultures by adapting a previously reported protocol (Shao et al., 2015). The cells of *B. subtilis* 168 were first grown in 2 ml 2xYT broth containing 10 μg/ml spectinomycin, 10 μg/ml erythromycin, and 5 μg/ml chloramphenicol to select the different constructs, respectively. The overnight cultures were inoculated into 2 ml fresh 2xYT broth without antibiotics and incubated at 37°C for 24 h, attaining full growth. The cultures were diluted 1:1,000 by fresh 2xYT broth without antibiotics and further incubated for 24 h where they were grown for the first 3 h at 37°C and then the temperature was reduced to 20°C to resemble the best conditions used for expression and production of taxadiene (growth of 1:1,000 dilution accounts for about 10 generations of cultivation, 2^10^ = 1,024). These cultures were diluted 10^6^ fold and plated onto 2xYT agar plates without antibiotics. Next day, 100 colonies were picked and transferred onto four different 2xYT agar plates supplemented with 10 μg/ml spectinomycin, 10 μg/ml erythromycin, 5 μg/ml chloramphenicol, or a combination of the three antibiotics. This treatment was successively repeated three times to obtain 30 generations of cultivation. The presence of the constructs was confirmed by the growth of the colonies on the antibiotic plates corresponding to the antibiotic resistance gene in each plasmid, thus indicating that the plasmid hosted by the colonies is segregationally stable. The whole experiment from beginning to end was performed in duplicate. The segregational stability of each construct was represented as the average of the % of colonies retaining the plasmid construct, which is equal to [colonies on 2xYT plate with antibiotic/colonies on 2xYT plate without antibiotic * 100%].

### Nucleotide Sequence Accession Number

The nucleotide sequence of the taxadiene synthase gene from the plant *T. baccata* was previously reported with the accession number: AY424738. The nucleotide sequence of the complete genome of *P. ananatis* was previously reported with the accession number: FUXY01000004. The *crtE* gene encoding the GGPPS enzyme was amplified from genomic DNA of *P. ananatis*, and the protein was assigned the accession number: SKA77365. The nucleotide sequence of the complete genome of *B. subtilis* 168 was previously reported with the following accession numbers: AL009126 and NC000964. The MEP pathway genes used in this study were amplified from the genomic DNA of *B. subtilis* 168.

## Results

### Construction of Different Strains of *B. subtilis* 168 Expressing Taxadiene Synthase

The first construct to be cloned was pDR_txs where the taxadiene synthase gene was cloned into pDR111 plasmid and then transformed into *B. subtilis*. This construct is designed to integrate the plant *txs* gene from *T. baccata* into the genome of *B. subtilis* where it is inserted into the *amyE* locus between the *amyE* front flanking region and *amyE* back flanking region (Overkamp et al., 2013). The *txs* gene expresses the enzyme taxadiene synthase, which is responsible for converting GGPP into taxa-4,11-diene. The expression of TXS from this construct is controlled by an engineered *B. subtilis mntA* ribosomal binding site and the strong IPTG inducible hyperspank promoter. The second construct produced was pBS0E_crtE, which is resulting from cloning the *crtE* gene from *P. ananatis* that encodes the GGPPS enzyme into pBS0E replicative plasmid. The GGPPS enzyme is responsible for the production of GGPP, which is the precursor of taxadiene. The pBS0E_crtE construct was transformed into *B. subtilis* strain containing pDR111_txs generating the txs + crtE strain. Finally, the construct overexpressing all genes of the MEP pathway along with *ispA* in one operon controlled by a xylose inducible promoter (p04_SDFHCEGA) was transformed into the txs + crtE strain generating the txs + crtE + SDFHCEGA *B. subtilis* strain. The aim was to increase the production of the precursors leading to the formation of taxadiene. All produced strains are listed in Table 2. The successful transformation of the constructs into the different *B. subtilis* strains was confirmed by colony PCR.

**Table 2:** *Bacillus subtilis* 168 strains generated in this study.

Strain	Constructs	Vectors	Genes in the operon
txs	pDR_txs	pDR111	*txs*
txs + crtE	pDR_txs pBS0E_crtE	pDR111 pBS0E	*txs* *crtE*
txs + crtE + SDFHCEGA	pDR_txs pBS0E_crtE p04_SDFHCEGA	pDR111 pBS0E pHCMC04G	*txs* *crtE* *dxs + ispD + ispH + ispF* + *ispC + ispE + ispG + ispA*

### Expression of Taxadiene Synthase in *B. subtilis* 168

**Figure 2:**
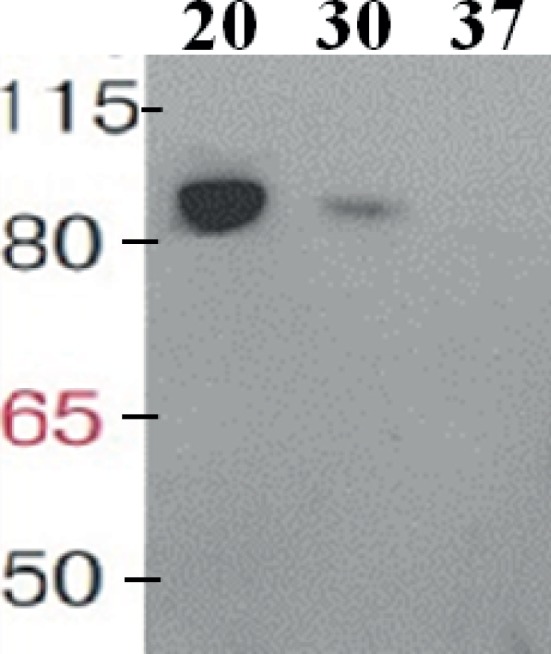
Western blot of the soluble protein fraction showing taxadiene synthase expressed in *Bacillus subtilis* 168 after integration into the chromosome using pDR111 plasmid. The lanes show TXS bands (~89 kDa) resulting from expression at different temperatures as follows: 20, 30, and 37°C. The enzyme was visualized by using specific antibodies against the His-tag.

The taxadiene gene used is the natural gene from the plant *T. baccata* and was combined with the *B. subtilis mntA* RBS and a N-terminal 6× His-tag. pDR111 plasmid was chosen to integrate the *txs* gene into the genome of *B. subtilis*. The cell culture was lysed, and in the soluble protein fraction, the expression of TXS (approximately 89 kDa) has been clearly detected on Western blot by using specific antibodies against the His-tag as shown in Figure 2. Different growth temperatures (37, 30 and 20°C) were tried to determine the best temperature for the expression of TXS using the pDR_txs construct following induction by IPTG. Since the samples from expression at different temperatures were loaded with the same total protein concentration on SDS-PAGE gel, a comparison of the TXS band can determine the temperature resulting in the best expression. A clear band representing TXS was seen on Western blot (Figure 2) following expression at 20°C, while a very faint band was the result of expression at 30°C and no TXS band was visible after expression at 37°C. Hence, the temperature that showed best TXS expression upon induction was 20°C. This will later be corroborated by the level of taxadiene production after incubation at the different temperatures.

### Level of Production of Taxadiene in Different Strains of *B. subtilis* 168

**Figure 3:**
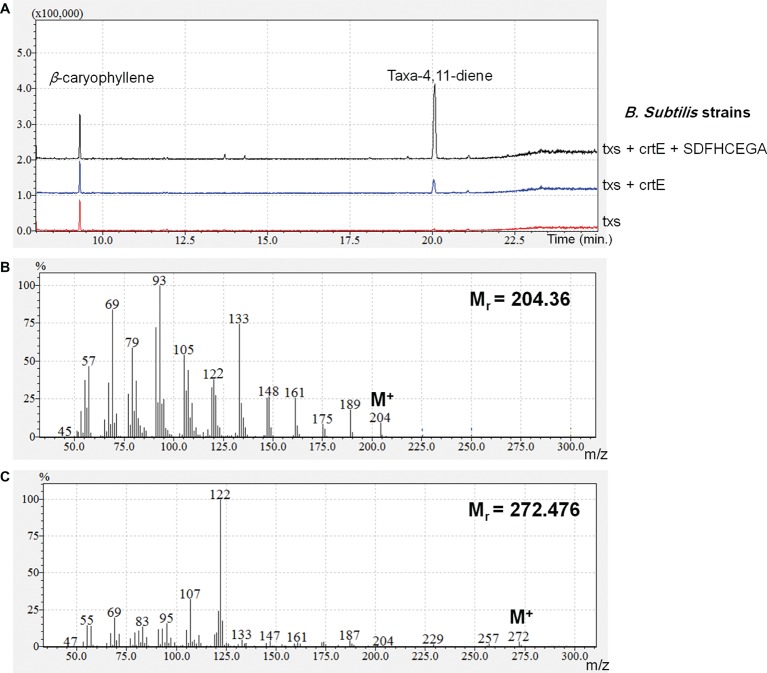
**(A)** GC chromatograms of the different strains of *Bacillus subtilis* in selected ion mode (SIM) for acquisition, monitoring m/z ion 122 to show the internal standard *β*-caryophyllene and the produced taxadiene peaks. **(B)** Mass spectrum of *β*-caryophyllene. **(C)** Mass spectrum of taxa-4,11-diene.

**Figure 4:**
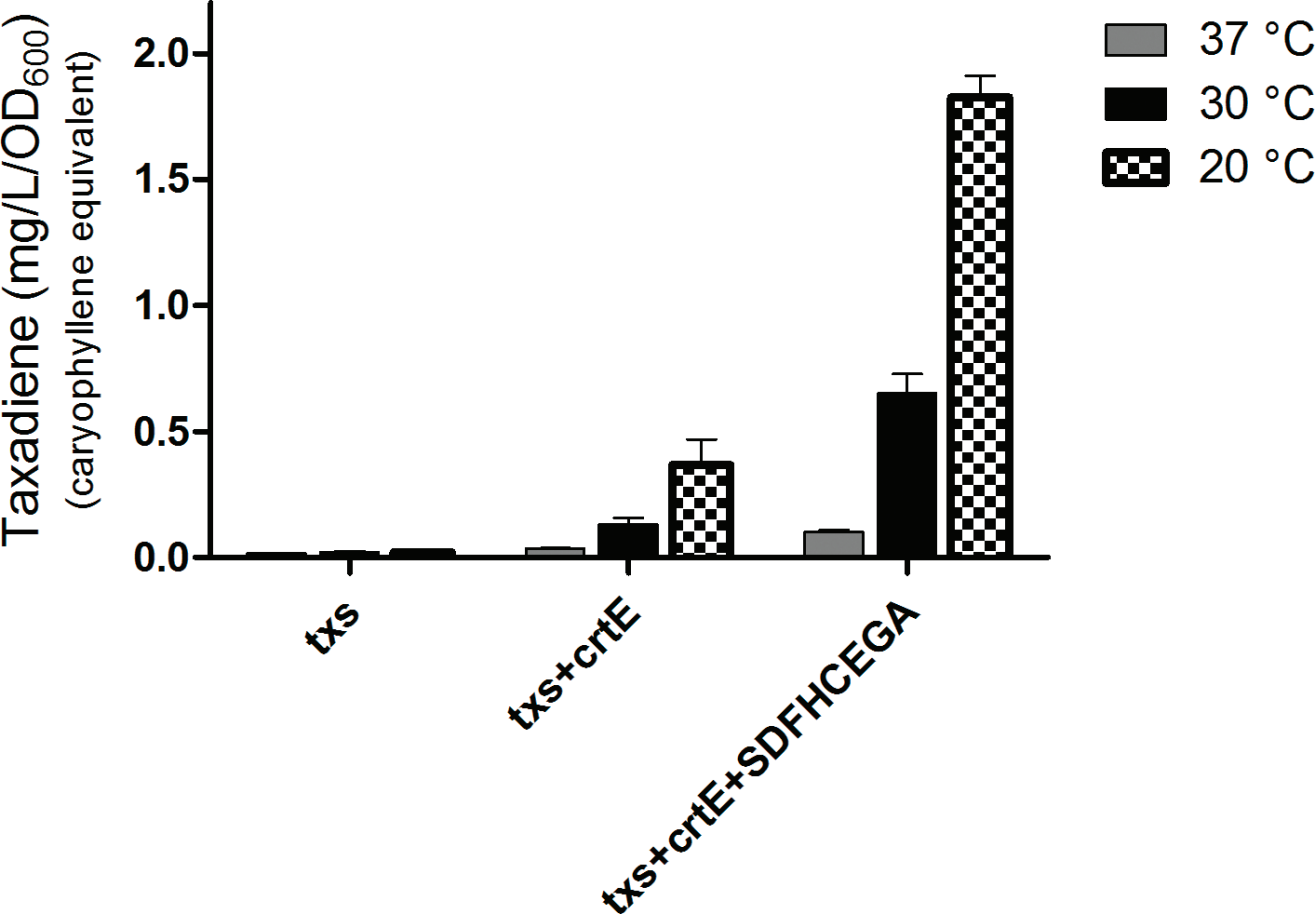
Amount of taxa-4,11-diene (mg/L/OD_600_) *β*-caryophyllene equivalent produced by the strains of *Bacillus subtilis* after incubation at 37, 30, or 20°C. The extraction of taxadiene was carried out without lysis of the cultures. The experiment was performed in triplicates.

**Figure 5:**
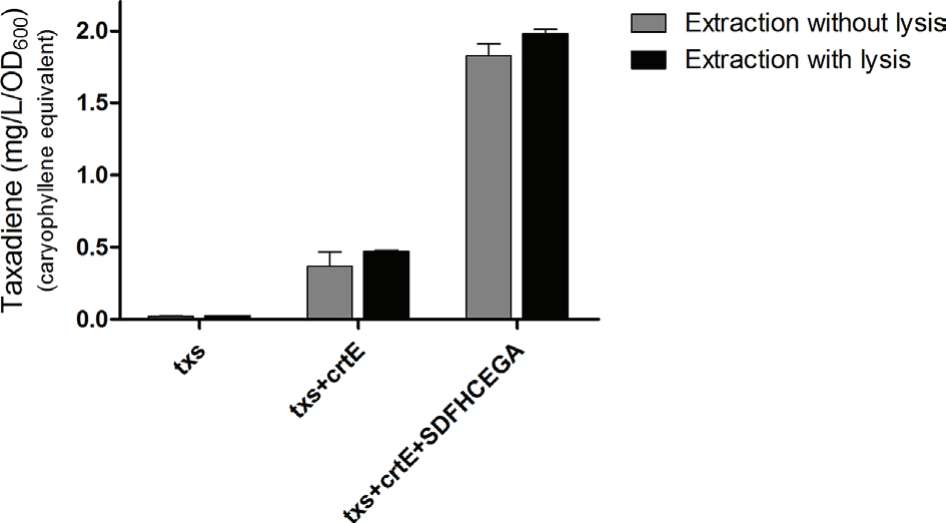
Amount of taxa-4,11-diene (mg/L/OD_600_) *β*-caryophyllene equivalent produced by the different strains of *Bacillus subtilis* after incubation at 20°C. A comparison of the amounts produced by extraction of taxadiene with and without lysis of the cultures is presented. The experiment was performed in triplicates.

The OD_600_ of the *B. subtilis* strains ranged from 7 to 9. The produced taxadiene was detected using GC-MS. The GC-MS chromatograms of the different *B. subtilis* strains showed the internal standard *β*-caryophyllene peak at retention time 9.25 min. and the taxadiene peak at retention time 20.05 min (Figure 3A). The SIM mode was used to monitor *m*/*z* ion 122 in the mass spectrum of both *β*-caryophyllene (Figure 3B) and taxadiene (Figure 3C). The peak areas were calculated and used to determine the concentration of produced taxadiene. The total amount of taxa-4,11-diene (mg/L/OD_600_) produced in the *B. subtilis* strains at the different incubation temperatures was compared (Figure 4). The highest level of taxadiene production was observed after incubation at 20°C, which coincides with the best expression of TXS. After that, the effect of extraction of taxadiene with and without cell lysis was also compared in the different strains when incubated at 20°C (Figure 5). The txs strain, which only contains the pDR_txs construct, relies on the innate MEP pathway, IspA (acting as GPPS and FPPS), and GGPPS for the production of taxadiene in *B. subtilis*. It showed the lowest production of taxadiene (0.024 mg/L/OD_600_) and was used as a control to compare the effect of overexpression of other genes on the production level. Introduction of *crtE* gene, encoding GGPPS enzyme, in the txs + crtE strain significantly increased the amount of taxadiene produced (0.471 mg/L/OD_600_), which is around 20 times higher than the control strain. This is probably due to the increased production of the precursor GGPP. Finally, combining the txs + crtE strain with overexpression of the MEP pathway genes and the *ispA* gene showed much higher amounts of taxadiene with the strain expressing all the genes showing the highest production levels (1.981 mg/L/OD_600_), which is approximately 83 times higher than the control strain. In addition, cell lysis before extraction did not show significant increase in the production levels compared to extraction without lysis (Figure 5). This indicates that taxadiene is released from the cells into the dodecane layer during growth. In fact, the omission of a cell lysis treatment resulted in a reduced contamination of the taxadiene due to the lysis buffer and SDS used. Actually, the non-lysed fermentation broth showed a thick cell pellet that could be removed easily by centrifugation, whereas the lysed broth showed a completely clear solution. Also, the chromatogram of the lysed culture showed extra peaks especially due to SDS. The addition of SDS was necessary for complete lysis, which could not be achieved with lysozyme alone. It is important to note that through following OD_600_ of the engineered strains compared to wild-type *B. subtilis*, it was clear that the engineered strains have slower growth rate especially at the best expression temperature which is 20°C. However, after the 24 h growth, both the engineered strains and wild type reached the same OD_600_.

### Segregational Stability of the Constructs in txs + crtE + SDFHCEGA *B. subtilis* Strain

**Figure 6:**
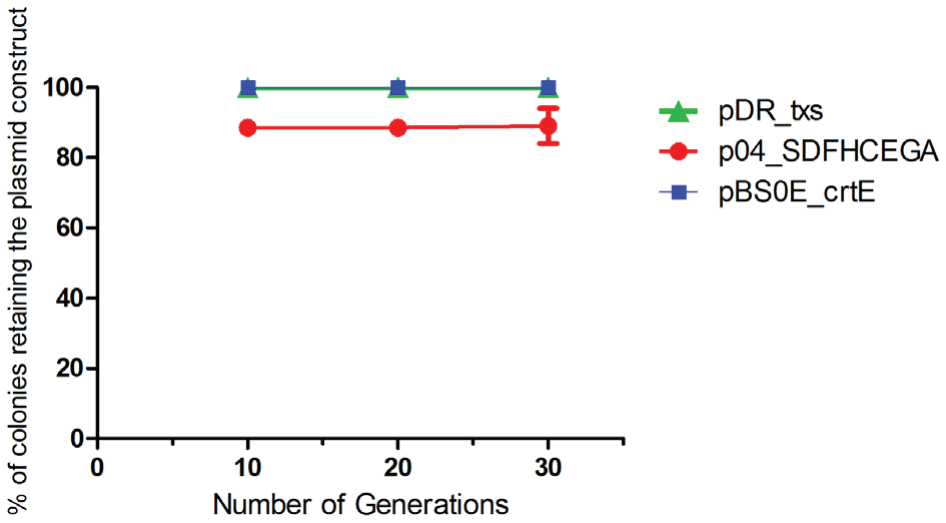
Segregational stability of pDR_txs, pBS0E_crtE, and p04_SDFHCEGA constructs in txs + crtE + SDFHCEGA *Bacillus subtilis* 168 strain. The stability is represented as the % of colonies retaining the plasmid constructs formed on the spectinomycin, erythromycin, or chloramphenicol-containing analytical plates, respectively, after successive subculturing in the absence of antibiotics (30 generations). The experiment was performed in duplicate.

A bacterial strain is considered segregationally stable when all daughter cells get at least one plasmid during cell division. The development of plasmid-free cells can lead to a significant loss in productivity. The segregational stability of the pDR_txs, pBS0E_crtE, and p04_SDFHECEGA constructs in the txs + crtE + SDFHCEGA *B. subtilis* strain was evaluated. The strain showed 100% ability to retain the pDR_txs and pBS0E_crtE constructs until the 30^th^ generation along with 90% ability to retain the p04_SDFHECEGA construct (Figure 6) in the absence of antibiotics. Thus, this stability should be sufficient for large-scale fermentations in the absence of antibiotics.

## Discussion

In the past few decades, researchers focused on the use of metabolic engineering and synthetic biology to manipulate a variety of hosts for biosynthesis of numerous terpenoids. Biosynthetic pathways of terpenoids have been studied, and the majority of the genes involved have been identified (Barkovich and Liao, 2001; Kirby and Keasling, 2008; Niu et al., 2017; Wang et al., 2017). Among these terpenoids is the chemotherapeutic paclitaxel. This study focused on the engineering of *B. subtilis* for the production of the first committed intermediate, taxa-4,11-diene, in the biosynthesis of paclitaxel. The biosynthesis of taxadiene in *B. subtilis* (Figure 1A) proceeds *via* the MEP pathway to produce IPP and DMAPP that would eventually be converted to the precursor GGPP. The enzyme taxadiene synthase cyclizes GGPP to produce taxa-4,11-diene (Croteau et al., 2006). Hence, the expression of the enzyme taxadiene synthase in *B. subtilis* is a prerequisite for the production of taxadiene. Moreover, tuning of the MEP pathway in *B. subtilis* has been previously explored (Guan et al., 2015; Xue et al., 2015) and can serve as a basis for improved production of taxadiene.

As a first step for the production of taxadiene in *B. subtilis*, the enzyme taxadiene synthase needs to be expressed. The gene encoding taxadiene synthase was amplified from the genome of the plant *T. baccata* that produces a protein of 862 amino acids. It has been reported that a pseudomature form of TXS where 60 amino acids were removed from the preprotein was superior in terms of level of expression, solubility, stability, and catalytic activity with kinetics analogous to that of the native enzyme (Williams et al., 2000). Hence, a truncated TXS protein eliminating the first 60 amino acids (plastid targeting sequence) was expressed in *B. subtilis*. For successful expression in *B. subtilis*, the *txs* gene along with the *B. subtilis mntA* RBS and N-terminal 6× His-tag was integrated into the chromosome using the pDR111 plasmid. The areas of the *amyE* gene flanking the *txs* gene in pDR111 allowed integration at the *amyE* locus in the *B. subtilis* chromosome. Following induction by IPTG at 20°C, a soluble TXS protein (~89 kDa) was expressed in *B. subtilis* and detected by Western blotting (Figure 2). The incubation at 20°C provided the best condition for expression of TXS as opposed to incubation at 30 or 37°C. The amount of taxadiene produced in this txs strain was very low, around 0.024 mg/L/OD_600_, indicating that further tuning of the pathway leading to the formation of the taxadiene precursor GGPP is essential to boost the taxadiene production level.

In pursuance of increasing the production of taxadiene, higher levels of the precursor GGPP is required. In the biosynthetic pathway (Figure 1A), IPP and DMAPP are converted to GPP with the enzyme GPPS, and then GPP is elongated to FPP using the enzyme FPPS, both encoded by the gene *ispA*. Finally, GGPPS enzyme produces GGPP from FPP. Studies on prenyl synthases of *B. subtilis* from the gene or protein perspective are still limited. *B. subtilis* is known for producing FPP, the precursor for sesquiterpenes, and triterpenes, through its *ispA* encoded GPPS and FPPS (Kunst et al., 1997; Kobayashi et al., 2003; Julsing et al., 2007; Barbe et al., 2009). The bacterium is also known to produce heptaprenyl (C_35_ terpenes) metabolites utilizing *hepT* and *hepS* encoding heterodimeric heptaprenyl diphosphate synthase enzymes that are responsible for producing heptaprenyl metabolite precursor from FPP (Zhang et al., 1998; Kontnik et al., 2008). Up until now, albeit it was reported that *B. subtilis* possesses an enzyme capable of producing GGPP from experiments with a semipurified protein of *B. subtilis* long before the genome sequence of *B. subtilis* was revealed (Takahashi and Ogura, 1981, 1982), the definite annotation of the gene encoding GGPPS is still uncertain. The gene that encodes GGPPS can be found in microorganisms that produce C_40_ carotenoids, such as lycopene, where GGPP precursor is needed in their biosynthesis. Examples of these microorganisms are the *Pantoea* genus with its *crtE* gene and *Corynebacterium glutamicum* with its *crtE* and *idsA* genes (Hara et al., 2012; Heider et al., 2014; Walterson and Stavrinides, 2015). *CrtE* of *Pantoea* has been widely used in metabolic engineering to produce high level of carotenoids and also C_20_ terpenoids in *E. coli* (Yoon et al., 2007). Hence, the *crtE* gene from *P. ananatis* was cloned in the pBS0E vector and transformed into the *B. subtilis* txs strain. The amount of taxadiene produced (Figure 5) by the txs + crtE strain (0.471 mg/L/OD_600_) is approximately 20 times higher than the control txs strain (0.024 mg/L/OD_600_). This indicates that overexpression of the GGPPS enzyme is essential to increase the formation of GGPP precursor in *B. subtilis* and in turn improve the production level of taxadiene.

Finally, overexpression of the MEP pathway leads to increased production of IPP and DMAPP. The *idi* gene (the final gene in the MEP pathway encoding for an IPP isomerase) was not overexpressed by us due to previous indications that it is non-essential in *B. subtilis* (Takagi et al., 2004). Past research also revealed that a knockout of the *idi* gene in *B. subtilis* is viable and produces isoprene, which implies that the synthesis of the isomer DMAPP does not rely on the isomerase (Eisenreich et al., 2004; Julsing et al., 2007). Note that, toxicity associated with the accumulation of prenyl diphosphates in *B. subtilis* with improved flux of MEP pathway has been reported (Sivy et al., 2011). Hence, it was important to control the p04_SDFHCEGA construct with an inducible promoter. The *B. subtilis* strain expressing TXS, GGPPS, all the MEP pathway, and IspA (acting as GPPS and FPPS) enzymes proved that high supply of the GGPP precursor is essential. The txs + crtE + SDFHCEGA strain showed the highest level of production of taxadiene (1.981 mg/L/OD_600_), which is approximately 83 times higher than the txs control strain. The OD_600_ of this strain was approximately 9; hence, the total production of taxadiene is around 17.8 mg/L. This production level is higher than the amount reported in yeast 8.7 mg/L (Engels et al., 2008) and in *E. coli* 11.3 mg/L (Bian et al., 2017) on shake flask fermentation level. Also, the taxadiene production in *B. subtilis* is comparable to amorphadiene production that was previously reported in *B. subtilis* (Zhou et al., 2013). Moreover, our strain showed segregational stability of the different constructs up to the 30^th^ generation of cultivation, which allow large-scale fermentations in the absence of antibiotics.

It is worth mentioning that the amount of taxadiene extracted from the cell culture with and without lysis is nearly the same (Figure 5). This indicates that taxadiene is released from the cells during growth and captured by the dodecane layer. It is also reported that the C_15_ amorphadiene produced in *B. subtilis* is trapped in the dodecane layer (Zhou et al., 2013) as we observed with our C_20_ taxadiene. Probably, due to their lipophilicity, they are able to permeate through the bacterial membrane and dissolve in the non-polar dodecane. However, C_30_ carotenoids are not exported from *B. subtilis* and have to be isolated from the cell pellet after lysis (Yoshida et al., 2009; Xue et al., 2015). Also, C_35_ heptaprenyl terpenes are produced in the spores of *B. subtilis* and not secreted (Bosak et al., 2008). The fact that taxadiene is not trapped in the cells and that lysis is not necessary, makes large-scale production much simpler where the *B. subtilis* cell culture in fermenters can be overlaid with dodecane to collect the released taxadiene. In addition, contaminants from the lysis solution can be avoided. As for *E. coli*, lysis is more likely to be needed due to its double membrane compared to *B. subtilis* which has a single membrane.

In conclusion, the successful expression of the enzyme taxadiene synthase in *B. subtilis* is reported for the first time. The expression of GGPPS in *B. subtilis* is crucial for the sufficient production of the essential precursor GGPP. Hence, the production level of taxadiene is boosted by overexpression of GGPPS enzyme along with MEP pathway and IspA (acting as GPPS and FPPS) enzymes. This is a proof of concept that taxadiene can be efficiently produced in *B. subtilis* and can serve as the basis for engineering *B. subtilis* as a cell factory for paclitaxel production. The txs + crtE + SDFHCEGA strain can be further engineered with additional enzymes (acyltransferases, cytochrome P450) necessary to produce 10-deacetylbaccatin III, which can be extracted and chemically converted to docetaxel or even paclitaxel (Walker and Croteau, 2000). In the future, efforts to improve titer such as optimization of the growth medium and/or deletion of competing pathways in *B. subtilis* that divert the flux of FPP or GGPP can be explored with caution to ensure avoiding pathways essential for the survival of the bacteria (Julsing et al., 2007; Guan et al., 2015; Zhao et al., 2016). Furthermore, process engineering to optimize pH, stirring, and aeration control will be required to develop the large-scale fed-batch fermentation system. Using the *B. subtilis* cell factory will eliminate the need to rely on the natural resources of the yew trees and thus avoid the endangering of the species and prevent shortfalls due to crop conditions.

## Author Contributions

IA and HP executed the experiments and interpreted the data. IA wrote the manuscript. HP revised the manuscript. RvM assisted with the experiments and revised the manuscript. S revised the data and manuscript. WQ conceived the project, confirmed the data, and revised the manuscript. All authors read and approved the final manuscript.

### Conflict of Interest Statement

The authors declare that the research was conducted in the absence of any commercial or financial relationships that could be construed as a potential conflict of interest.
